# Structure of the Fab fragment of a humanized 5E5 antibody to a cancer-specific Tn-MUC1 epitope

**DOI:** 10.1107/S2059798325002554

**Published:** 2025-04-13

**Authors:** Wei Li, Ulla Mandel, Henk van Faassen, Matthew J. Parker, Max S. G. Legg, Greg Hussack, Henrik Clausen, Stephen V. Evans

**Affiliations:** a Department of Biochemistry and Microbiology University of Victoria PO Box 3055 STN CSC Victoria BCV8P 3P6 Canada; b Copenhagen Center for Glycomics, Department of Cellular and Molecular Medicine, Faculty of Health Sciences University of Copenhagen Blegdamsvej 3 2200Copenhagen N Denmark; c Human Health Therapeutics Research Centre National Research Council Canada 100 Sussex Drive Ottawa ONK1A 0R6 Canada; McGill University, Canada

**Keywords:** cancer, anti-Tn-MUC1 antibody, 5E5, Tn antigen, X-ray crystallography, humanized antibodies, Fab structure

## Abstract

The structure of the humanized Fab from murine monoclonal antibody 5E5 specific for tumor antigen Tn-MUC1 has been determined to 1.57 Å resolution. Despite undertaking thousands of crystallization trials of the humanized 5E5 (h-5E5) Fab in the presence of either the singly or doubly glycosylated peptide antigens corresponding to Tn-MUC1, the Fab is only observed unliganded in the crystal. The conformations of the complementarity-determining regions (CDRs) of the combining site on the h-5E5 Fab do not differ significantly from those reported for liganded murine scFv at 3.0 Å resolution. While the affinity of the murine 5E5 has previously been reported as *K*_D_ = 1.7 n*M* for the 24-mer Tn-MUC1 peptide PPAHGVT*SAPDTRPAPGS*T*APPAH prepared by *in vitro* glycosylation of a synthetic 24-mer MUC1 peptide, the *K*_D_ of the h-5E5 Fab for the shorter doubly glycosylated glycopeptide antigens PAPGS*T*AP and APGS*T*AP was measured here as only 41 and 61 µ*M*, respectively. Interestingly, the single Fab molecule in the asymmetric unit of space group *C*2 is observed packed head-to-head with a symmetry-related Fab across a crystallo­graphic twofold axis such that a polypeptide loop from the light chain of each Fab is observed to insert into the antigen-binding pocket of the symmetry-related Fab. While this might suggest that binding of the Tn-MUC1 peptides may have been inhibited by a homophilic association, none was detected. The humanization process has imposed changes in the framework regions of the Fv which may have affected the Vh–Vl interface.

## Abstract 2

The structure of the humanized Fab from murine monoclonal antibody 5E5 specific for tumor antigen Tn-MUC1 has been determined to 1.57 Å resolution. The humanization process has imposed changes in the framework regions of the Fv which may have affected the Vh–Vl interface.

## 1. Introduction

Mucins are heavily glycosylated polypeptide chains that form the backbone of mucus, with generally more than half of their mass consisting of O-linked glycans (Johansson & Hansson, 2016 ▸). The human mucin family has 21 members (Kufe, 2009 ▸), among which mucin 1 (MUC1) is a transmembrane heterodimer that consists of an extracellular N-terminal subunit (MUC1-N) and a C-terminal subunit (MUC1-C) (Andrulis *et al.*, 2014 ▸; Kufe, 2008 ▸). MUC1-N mainly consists of variable-number (25–125) tandem repeats (VNTRs) of a stretch of 20 highly conserved amino-acid residues: HGVTSAPDTRPAPGSTAPPA. Each VNTR has five potential sites for O-glycosylation and MUC1-N subunits are normally heavily glycosylated (Tarp *et al.*, 2007 ▸).

MUC1 chains are highly overexpressed and aberrantly O-glycosylated (Taylor-Papadimitriou *et al.*, 2018 ▸) on the cell surfaces of a number of different cancers, with variations in the density and length of glycan determinants that result in the formation of tumor-associated carbohydrate antigens such as Thomsen–Friedenreich (T) antigen and Thomsen nouvelle (Tn) antigen, and their sialylated forms ST and STn, respectively (Soliman *et al.*, 2017 ▸; Julien *et al.*, 2009 ▸). These changes serve to distinguish tumor-associated MUC1s from that of normal cells (Yin *et al.*, 2018 ▸), rendering them as targets for antibodies and lectins.

Aberrant glycosylation patterns are common to many types of cancer (Beatson *et al.*, 2016 ▸), and in 2009 MUC1 was ranked number 2 of 75 tumor-associated antigens as cancer-vaccine targets by the National Cancer Institute Translational Research Working Group (Zhou *et al.*, 2018 ▸). The Tn-MUC1 antigen has been reported to be expressed in 70–90% of breast, lung, prostate and pancreatic tumors (Taylor-Papadimitriou *et al.*, 2018 ▸) and there is a direct positive correlation between the prognosis of cancer and occurrence of the Tn antigen (Rømer *et al.*, 2021 ▸), making the Tn antigen a prime target for cancer diagnosis and immunotherapy (Tarp *et al.*, 2007 ▸; Burchell *et al.*, 1987 ▸).

A number of mAbs have been raised targeting the glycosylated and unglycosylated VNTR regions of MUC1. The mAb ‘stripped mucin 3’ (Ab SM3) recognizes the unglycosylated sequence PDTRP in the VNTR region (Dokurno *et al.*, 1998 ▸). Antibodies HMFG1, HMFG2, EMA and 5E10 all react with the MUC1 peptide backbone, with some dependence on the glycosylation state (Lavrsen *et al.*, 2013 ▸; Andrulis *et al.*, 2014 ▸; Burchell *et al.*, 1987 ▸), while EMA has a higher binding affinity for the glycosylated form (Andrulis *et al.*, 2014 ▸). Glycosylation is observed to affect the binding of most of these mAbs to varying degrees.

Murine mAbs 5E5 and 2D9 have a similar specificity, both targeting Tn-MUC1 in the GSTAP region; 2D9 requires *N*-acetylgalactosamine (GalNAc) to be O-linked to both Ser and Thr in GSTAP, while 5E5 can bind GSTAP when it is only singly O-linked to GalNAc (Lavrsen *et al.*, 2013 ▸; Tarp & Clausen, 2008 ▸; Posey, Clausen *et al.*, 2016 ▸; Sørensen *et al.*, 2006 ▸). MAb 5E5 has a relatively high affinity for Tn-MUC1 and has been observed to induce antibody-dependent cellular cytotoxicity (Lavrsen *et al.*, 2013 ▸). It has a higher binding affinity for Tn-MUC1 (*K*_D_ = 1.7 n*M*) compared with that for STn-MUC1 (*K*_D_ around 100 n*M*) (Lavrsen *et al.*, 2013 ▸; Kračun *et al.*, 2010 ▸). Murine 5E5 reacts with the vast majority of breast carcinomas, yet shows no observable reactivity with normal breast epithelial cells, and induces antibody-dependent cellular cytotoxicity but not complement-dependent cytotoxicity in breast cancer cell lines T47D and MCF-7 (Lavrsen *et al.*, 2013 ▸; Taylor-Papadimitriou *et al.*, 2018 ▸). Chimeric antigen receptor (CAR) T cells designed with 5E5 single-chain fragment variable (scFv) targeting the Tn-MUC1 antigen have been reported to control tumor growth in xenograft models of T-cell leukemia and pancreatic cancer (Posey, Schwab *et al.*, 2016 ▸) and to eliminate intrahepatic cholangiocarcinoma cells (Mao *et al.*, 2023 ▸). Thus, humanized 5E5 (h-5E5) is a candidate for development as a passive immunotherapy (Lavrsen *et al.*, 2013 ▸).

In order to reduce the immunogenicity of the murine 5E5 mAb while retaining specificity and binding affinity, humanized anti-Tn-MUC1 Abs were generated based on the murine 5E5 mAb. 29 variable domains of the heavy chains (Vhs) were used (Supplementary Fig. S1) corresponding to human Ab sequences (SEQ ID Nos. 1–29, where No. 1 is mouse) and five variable domains of the light chains (Vls; SEQ ID Nos. 30–34, where No. 30 is mouse; Van Berkel *et al.*, 2017 ▸). The constant domains utilize the consensus sequences of human IgG1κ (Van Berkel *et al.*, 2017 ▸). The mutations of the various Vhs and Vls were produced by insertions, substitutions or deletions which maintained the ability to bind to the Tn-MUC1 antigen, and the maximum number of such variations turned out to be 20 (Van Berkel *et al.*, 2017 ▸). With each of the 29 different sequences of the Vhs paired with five different sequences of the Vls, a total of 145 possible humanized mAbs could be generated, with one of them being the murine 5E5 (SEQ ID No. 1 for Vh and SEQ ID No. 30 for Vl). According to Van Berkel *et al.* (2017 ▸), some of the humanized mAbs generated demonstrated improved affinity for the Tn-MUC1 antigen.

The structures of four MUC1-targetting Abs have been reported in complex with their respective glycosylated antigens; the first was SM3, which recognizes PDTRP (PDB entry 1sm3; Dokurno *et al.*, 1998 ▸), the second was AR20.5, which bound to DTRPAP in the VNTR region (PDB entries 5t78 for the glycopeptide and 5t6p for the peptide; Movahedin *et al.*, 2017 ▸), the third was SN-101 in complex with VTSAPDT*RPA (PDB entry 6kx1; Wakui *et al.*, 2020 ▸) and the fourth was the 3.0 Å resolution structure of the scFv of the murine 5E5 (scFv-5E5) reported in complex with the ligand APGST*AP (PDB entry 6tnp; Macías-León *et al.*, 2020 ▸). Although the reported structure displayed electron density corresponding to a fragment of the singly glycosylated peptide located in the Ab combining site, the relatively low reported resolution lends some ambiguity to the precise structure of the epitope.

In an effort to solve the structure of the paratope to higher resolution and to determine what, if any, changes in structure were induced in the Ab during the humanization process, the structure of the unliganded Fab from h-5E5 has been determined to 1.57 Å resolution.

## 2. Materials and methods

### 2.1. Fab preparation and purification

The humanized anti-Tn-MUC1 mAb was designed from 5E5 and was provided by ADC Therapeutics (Van Berkel *et al.*, 2017 ▸). The Fabs were prepared by papain digestion, with the optimal conditions observed to be a papain:IgG ratio of 1:200(*w*:*w*) at room temperature with a total digestion time of 5 h in 20 m*M* HEPES pH 7.5, 1 m*M* DTT. Iodoacetamide (10 m*M*) was used to quench the reaction. The reaction mixture was dialyzed overnight (20 m*M* HEPES pH 6.5). Undigested Ab and Fc fragments were removed using cation-exchange HPLC. The Fab fragment was dialysed overnight at 4°C against 20 m*M* MES pH 5.8, 100 m*M* NaCl.

### 2.2. Crystallization

The Fab of the humanized anti-Tn-MUC1 mAb was concentrated to 8 mg ml^−1^ using Amicon concentrators. Initial crystallization trials were performed using the vapor-diffusion method with the commercial screens NeXtal PEGs Suite, NeXtal PEGs II Suite, Index (Hampton Research) and JCSG+ (Molecular Dimensions) in sitting-drop 96-well Intelli-Plates (Hampton Research) using a Crystal Gryphon (Art Robbins Instruments, Sunnyvale, California, USA). The best crystals were obtained in 24% PEG 4000, 0.2 *M* sodium acetate, 0.1 *M* Tris pH 8.5 in subsequent optimization steps using the hanging-drop vapor-diffusion method at 19°C. It took two days for crystals to appear and four days for them to reach full growth.

### 2.3. Structure solution and refinement of h-5E5 Fab

Crystals were protected in mother liquor supplemented with 20% MPD and flash-cooled in a nitrogen stream at 100 K (Crystal Cooler) for data collection. Diffraction was observed to 1.57 Å resolution on Rigaku MicroMax-007 HF microfocus rotating-anode X-ray generator equipped with a Dectris PILATUS3 R 200K-A detector system at a wavelength of 1.5418 Å. X-ray data were processed using *HKL*-2000. The crystals were monoclinic and belonged to space group *C*2. The data-collection parameters are given in Table 1 ▸. The crystals appeared to be stable in the mother liquor stored at 19°C.

The structure of the humanized Fab was solved by molecular replacement with *Phaser* (McCoy *et al.*, 2007 ▸) using the scFv of 5E5 (PDB entry 6tnp; Macías-León *et al.*, 2020 ▸) as a search model for the variable domains Vh and Vl and the Fab of an anti-CD40 Ab, ABBV-323 (PDB entry 6pe7; Argiriadi *et al.*, 2019 ▸), as a search model for the constant domains CH1 and CL, and was refined using *REFMAC*5 and *Phenix*. Refinement statistics are listed in Table 1 ▸.

The amino-acid sequence was changed from the molecular-replacement model to that of the humanized mAb using *CCP*4 (Agirre *et al.*, 2023 ▸), with the correct sequence established by comparison of the electron-density map at high resolution with those of the 145 humanized mAbs in the pool from which it was generated (Van Berkel *et al.*, 2017 ▸). With data to 1.57 Å resolution, no difficulty was encountered in selecting the correct amino-acid sequence, which was determined to be sequence 5 (SEQ ID No. 5); however, there was unambiguous electron density at position 48 for a methionine residue (Supplementary Fig. S2) instead of the indicated isoleucine. Overall, the identities of 22 of the 116 residues in the heavy chain were changed on going from the murine to the humanized sequence, as were 15 of the residues in the light chain. All of the mutations lie at or near the surface of the protein and are distal from the combining site.

### 2.4. Ab–antigen binding analysis for h-5E5 Fab with doubly glycosylated Tn-MUC1 peptides using surface plasmon resonance

The binding kinetics of the h-5E5 Fab to doubly glycosylated Tn-MUC1 peptides were studied by surface plasmon resonance (SPR) using a Biacore T200 instrument (GE Healthcare). All experiments were performed at 25°C with HBS-EP (10 m*M* HEPES, 150 m*M* NaCl, 3 m*M* EDTA, 0.005% Tween 20 pH 7.4) as the running buffer. The purified h-5E5 Fab and an irrelevant 1104 Fab were immobilized [∼10 000 resonance units (RUs) each] on a Series S Sensor Chip CM7 (Cytiva) using an amine-coupling kit (Cytiva) in 10 m*M* acetate buffer pH 4.0 (Cytiva). Serial dilutions of doubly glycosylated Tn-MUC1 peptides in HBS-EP buffer were injected at a flow rate of 20 µl min^−1^ over flow cells containing immobilized h-5E5 Fab and the 1104 Fab (as a negative control). The reference flow cell was blocked with ethanolamine. The association time was set to 30 s and the dissociation time to 60 s. After each run, the sensor chip was regenerated with HBS-EP buffer. The concentrations of the analytes injected were 6.25, 12.5, 25, 50 and 100 µ*M*. The analytes were two doubly glycosylated Tn-MUC1 peptides, PAPGS*T*AP and APGS*T*AP, where * represents GalNAc. Reference flow cell subtracted sensorgrams were analyzed with *BIAevaluation Software* v.3.2 (Cytiva) and affinities were determined by steady-state analysis from three experimental replicates. PAPGS*T*AP binding to amine-coupled 5E5 mAb (∼25 000 RUs) was determined under the same conditions described for the Fab.

### 2.5. Self-interaction analysis of the h-5E5 Fab using SPR

Fab–Fab self-interactions for the h-5E5 Fab were analyzed using SPR, along with the 1104 Fab as a comparator. All experiments were performed at 25°C in HBS-EP running buffer. The purified h-5E5 Fab and control 1104 Fab were immobilized on a Series S Sensor Chip CM7 (Cytiva) as described above to ∼10 000 RUs. Serial dilutions of the Fabs were injected at a slow flow rate of 10 µl min^−1^ with a prolonged contact time over the Fab surfaces for 1800 s. The dissociation time was 900 s. The concentrations of the Fabs injected were 1.5, 3, 6, 12 and 24 µ*M*. Similar self-interaction SPR experiments were attempted with 5E5 mAb and control mAb 324, in which ∼25 000 RUs were immobilized on a CM7 chip and mAbs were flowed up to 10 µ*M*.

Attempts were also made to decrease the salt concentrations to 25 m*M* (from 150 m*M*) but both Fabs started to show considerable nonspecific binding to the dextran layer on the sensor chip.

The purity and stability of the Fabs were confirmed prior to all SPR experiments using a size-exclusion chromatography column (Superdex S200 Increase 10/300 GL, Cytiva) on an ÄKTApure chromatography system (GE Healthcare) using HBS-EP running buffer at a flow rate of 0.8 ml min^−1^. Both Fabs had symmetrical, monodisperse profiles indicative of highly pure monomers devoid of aggregates (data not shown).

## 3. Results

### 3.1. Amino-acid sequence determination

The manner in which the h-5E5 Fab was generated pre­cluded determination of the gene sequence. Specifically, the Fv domains were constructed from a library of multiple redundant gene segments and the mixture was panned against Tn-MUC1, with the humanized construct displaying the greatest affinity selected in any panning step. Indeed, some humanized clones were reported to have higher affinity for the antigen than the murine Ab (Van Berkel *et al.*, 2017 ▸). As such, the gene sequences generated by the selected constructs were not known directly, and the amino-acid sequence had to be estimated instead from a residue-by-residue comparison of the published possible sequences from Supplementary Fig. S1 with the observed electron densities. Fortunately, the process was made straightforward by the high resolution of the X-ray diffraction data collected (a summary of X-ray diffraction data-collection statistics is given in Table 1 ▸), and the sequences of the heavy and light chains were quickly deduced. For the sequence of the heavy-chain variable region, sequence SEQ ID No. 5 (Van Berkel *et al.*, 2017 ▸) was found to match the electron density most closely, and for the light-chain variable region the sequence SEQ ID No. 31 (Van Berkel *et al.*, 2017 ▸) was found to match best. The sequences used for the constant regions were assigned from the consensus sequences from human IgG1κ, which also corresponded well to the observed electron density.

Overall, the reported process was designed to limit the total number of mutations on each chain to 20 (Macías-León *et al.*, 2020 ▸). While 15 mutations were found on the light chain, the number was somewhat higher on the heavy chain, with 22 mutations. Interestingly, one unintended mutation was discovered in the observation of unambiguous electron density for methionine for residue 48 of the heavy chain (Met-H48) that was designed to be isoleucine (Macías-León *et al.*, 2020 ▸; Supplementary Fig. S2). (Ile-H48-Met involves a single point mutation in the third base of the codon.) The point substitution of methionine for isoleucine has been reported to lead to only minor changes in hydrophobicity (Ohmura *et al.*, 2001 ▸); however, while the corresponding C^α^ atom is ∼10 Å from the combining site and the side chain points away, it has nevertheless been known for some time that amino-acid mutations as far as 15 Å from the combining site can affect binding (Wedemayer *et al.*, 1997 ▸).

As designed from the humanization process, all of the planned sites of amino-acid residue mutation (Van Berkel *et al.*, 2017 ▸) lie at or near the surface of the Fab where they are exposed to immune surveillance, and while most are distal from the combining site a few come as close as 7 Å.

### 3.2. Quality of the model

The h-5E5 Fab crystallized in the monoclinic space group *C*2, and it shows continuous electron density along almost all of the polypeptide chain. The exceptions are residues 131–135 at the ‘bottom’ of the constant domain of the Fab heavy chain, the final three C-terminal residues of the heavy chain and the C-terminal residue of the light chain, which were not modeled due to lack of electron density. No φ–ψ angles are observed to lie in disallowed regions, with 98.1% in favored regions and 1.9% in allowed regions (using *MolProbity* from the *Phenix* graphical user interface; Liebschner *et al.*, 2019 ▸).

The high resolution of the structure allowed multiple conformations to be modeled for some of the side chains, including Arg-H74, Ser-H75, Met-H93 and Met-H111. A total of 768 water molecules were modeled in the final structure, as was one clear example of the cryoprotectant MPD.

Having determined the structure of the Fab from crystals grown in the presence of antigen to 1.57 Å resolution, there was only an ambiguous lump of electron density in the combining site that was insufficient to convincingly model any part of the antigen.

### 3.3. The Fabs crystallize in a head-to-head arrangement

**Figure 1:**
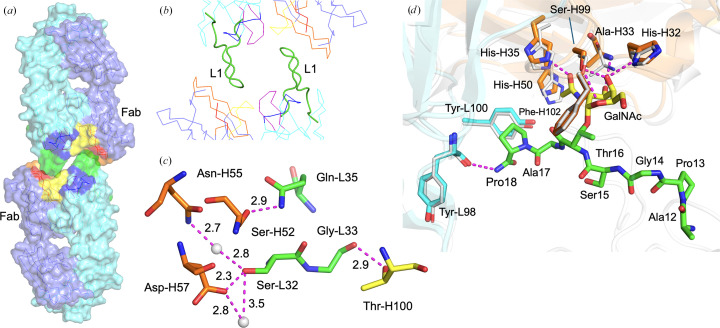
(*a*) A surface representation of two Fabs related by a crystallographic twofold axis shows that their L1 loops extend into pockets normally expected to bind antigen on the other Fab. Light chain, cyan; heavy chain, slate. (*b*) Close-up of the interaction with L1 shown as a ribbon and the remaining CDRs as wireframe. (*c*) Bonding interactions between the L1 loop of one Fab and its neighboring Fab. (*d*) Least-squares superposition of the C^α^ atoms of the Fv from the Fab of the h-5E5 mAb with molecule II of the murine scFv (including the observed position of the APGST*AP ligand in molecule II), with the heavy- and light-chain amino-acid residues that bind the ligand labeled and shown using the stick representation in *PyMOL*, and with the remainder of both Fvs shown as cartoon shadows. It is apparent that changes in heavy–light chain domain association have led to significant differences in the relative positions of corresponding antigen-binding residues to the extent that the binding of antigen would be expected to be affected. H-5E5, white; scFv-5E5 heavy chain, light brown; scFv-5E5 light chain, light blue. The hydrogen bonds between the ligand and mouse scFv-5E5 are shown as dashed lines. CDRs are labeled. H1, red; H2, orange; H3, yellow; L1, green; L2, blue; L3, purple. Water molecules, gray.

The h-5E5 Fab displays the familiar immunoglobulin fold, with the antigen-binding site made of six CDRs: three from the heavy chain, H1, H2 and H3, and three from the light chain, L1, L2 and L3, with an unusually long L1 (Fig. 1 ▸). Fabs are generally observed packed head-to-tail in the crystal lattice (Cygler *et al.*, 1987 ▸; Ban *et al.*, 1996 ▸), where the variable domains of one molecule interact with the constant domains of a neighboring molecule. The h-5E5 Fab is observed to pack head-to-head about a crystallographic twofold axis that serves to project the L1 loop of the Fab at (*x*, *y*, *z*) into the combining site of the Fab at (−*x*, *y*, −*z*) near the putative site of antigen binding and vice versa (Figs. 1 ▸*a* and 1 ▸*b*), where they form several strong hydrogen bonds directly linking the pair of Fabs (Table 2 ▸). This intimate contact observed between combining sites in the crystal lattice led to an investigation of the potential for homophilic binding in h-5E5 mAb.

### 3.4. SPR shows that both glycopeptides display relatively weak binding to amine-coupled h-5E5 Fab

**Figure 2:**
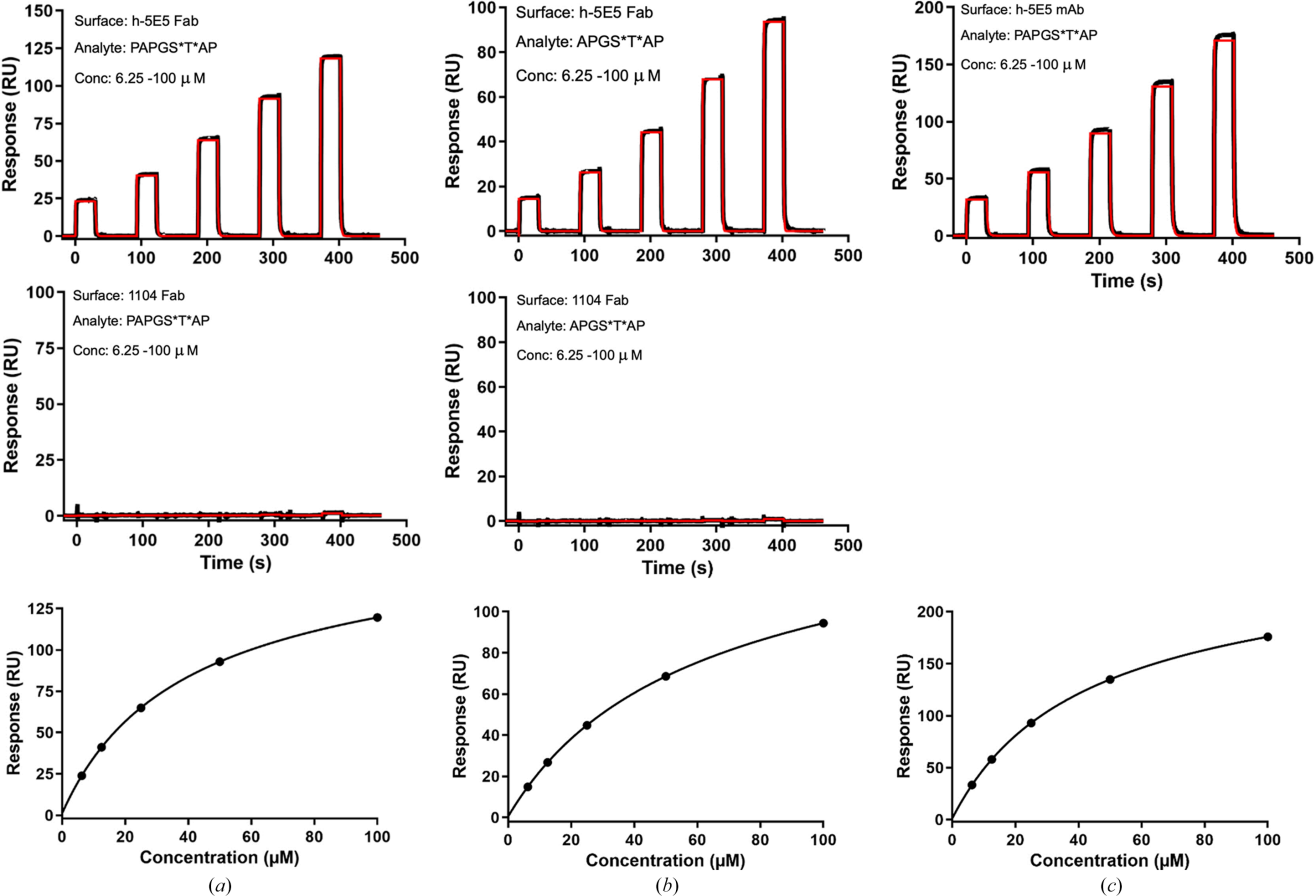
(*a*) Top: representative SPR sensorgram for the binding analysis of h-5E5 Fab with PAPGS*T*AP. Middle: the unrelated 1104 Fab served as a control surface. Fabs were amine-coupled and glycopeptide-flowed. Bottom: steady-state plot used to determine the h-5E5 binding affinity (*K*_D_). (*b*) Top: representative SPR sensorgram for the binding analysis of h-5E5 Fab with APGS*T*AP. Middle: the unrelated 1104 Fab served as a control surface. Fabs were amine-coupled and glycopeptide-flowed. Bottom: steady-state plot used to determine the h-5E5 binding affinity (*K*_D_). (*c*) Top, representative SPR sensorgram for the binding analysis of 5E5 mAb with PAPGS*T*AP. The mAb was amine-coupled and glycopeptide-flowed. Bottom, steady-state plot used to determine the 5E5 mAb binding affinity (*K*_D_).

Binding curves for both glycopeptides (PAPGS*T*AP and APGS*T*AP) against immobilized h-5E5 Fab showed good fits to a steady-state binding model, with *K*_D_ values of 40.7 ± 0.1 and 60.9 ± 0.1 µ*M*, respectively (Figs. 2 ▸*a* and 2 ▸*b*). The observed *R*_max_ of 165 RU (70% of the theoretical *R*_max_) for PAPGS*T*AP and 150 RU (70% of the theoretical *R*_max_) for APGS*T*AP indicates that the binding activity of both glycopeptides was high. Neither glycopeptide bound to the control surface, the amine-coupled Fab 1104, showing that the binding of both glycopeptides was specific to h-5E5 Fab. Binding of PAPGS*T*AP to immobilized h-5E5 mAb showed a similar affinity (*K*_D_ = 43.9 µ*M*) to that of the h-5E5 Fab (Fig. 2 ▸*c*).

### 3.5. SPR did not reveal any significant homophilic binding

**Figure 3:**
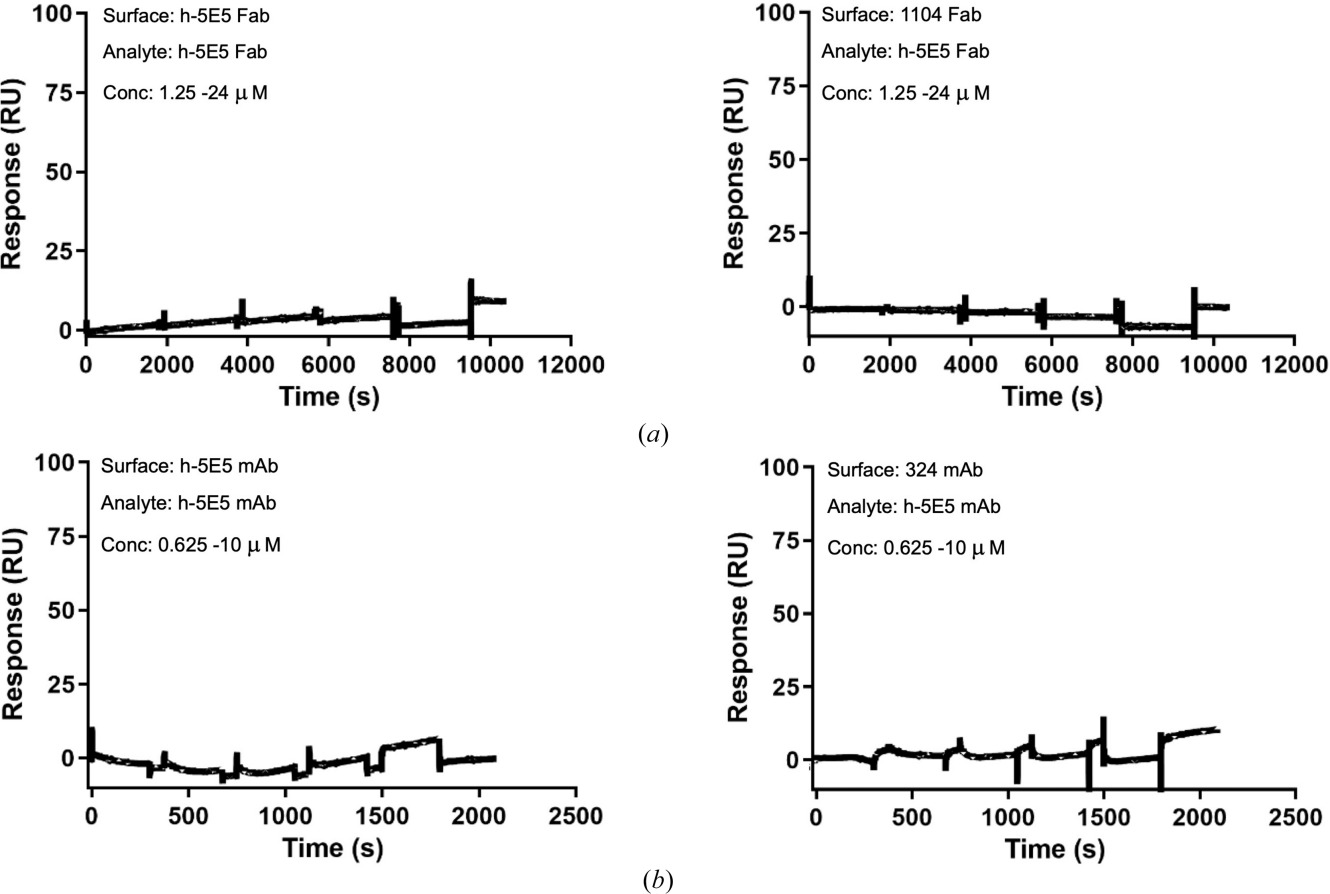
SPR sensorgrams demonstrating the absence of self-interaction of h-5E5 Fab (*a*) and h-5E5 mAb (*b*). Fabs [h-5E5 (left) and 1104 control (right) in (*a*)] were amine-coupled at a high density (∼10 000 RUs) and h-5E5 Fab flowed aup to 24 µ*M*. MAbs [h-5E5 (left) and 324 control (right) in (*b*)] were amine-coupled at a high density (∼25 000 RUs) and h-5E5 mAb flowed up to 10 µ*M*.

Despite the observation of a close association of pairs of h-5E5 Fab in the crystal lattice through their combining sites, binding curves for high-density surfaces of immobilized h-5E5 Fab against h-5E5 Fab (Fig. 3 ▸*a*) and immobilized h-5E5 mAb against h-5E5 mAb (Fig. 3 ▸*b*) show that no Fab–Fab self-interaction was observed for either the h-5E5 Fab or the h-5E5 mAb. The theoretical *R*_max_ for these self-interaction experiments was high (∼10 000 RU, corresponding to ∼10 ng mm^−2^ or ∼2 m*M* Ab concentrations on the sensor chip), and analytes (Fab or mAb) were flowed up to 24 µ*M* without a trace of binding.

## 4. Discussion

In a process first reported decades ago (Riechmann *et al.*, 1988 ▸), potential therapeutic mAbs from xenogeneic sources have been humanized (*i.e.* mutating amino-acid residues on the exterior of the mAb to correspond to their human counterparts) in order to preclude or impede the mounting of the host immune defense. A number of humanized therapeutic mAbs have successfully been developed, including emicizumab (Kitazawa *et al.*, 2017 ▸), a bispecific mAb that targets activated coagulation factors IX and X for the treatment of hemophilia A; cetuximab, which targets epidermal growth factor receptor (EGFR) for the treatment of metastatic colorectal cancer and head and neck cancer; and trastuzumab, which targets human epidermal growth factor receptor 2 (HER2; Zahavi & Weiner, 2020 ▸) for the treatment of breast cancer.

The process of humanization encompasses a wide swathe of techniques that range from simple replacement of the foreign constant regions of the Ab with human constant regions to form ‘chimeric’ antibodies (Morrison *et al.*, 1984 ▸) to the generation of transgenic animals with genomes that code for antibodies using human genes (Houdebine, 2002 ▸). The 5E5 mAb was humanized from the original murine mAb specific for the immunodominant GSTAP glycopeptide epitope in the MUC1 tandem repeat (Tarp *et al.*, 2007 ▸) by the mutation of surface residues on the framework regions of the variable domain dimer and the use of human subclass IgG1 and κ constant regions for the heavy and light chains, respectively (Van Berkel *et al.*, 2017 ▸). This report represents the first structural characterization of the humanized Fab based on murine anti-Tn-MUC1 mAb 5E5.

### 4.1. The Fab from h-5E5 displays self-association in the crystal

An interesting feature in the combining site of h-5E5 Fab is the protruding CDR L1 (comprised of 17 amino acids KSSQSLLNSGDQKNYLT; Figs. 1 ▸*a* and 1 ▸*b*), which reaches into the combining site of a neighboring molecule in the crystal lattice to form six strong hydrogen bonds (Fig. 1 ▸*c* and Table 2 ▸), which may have served to exclude the antigen from the combining site. In addition, the side chain of Ser-L32 of the CDR L1 interacts with Asn-H55 and Asp-H57 from the same symmetry-related Fab through bridging water molecules. While the residues involved in forming hydrogen bonds are not the same ones used to bind antigen in the murine Fv structure, the loops from the neighboring Fab do occlude the putative antigen combining site (Supplementary Fig. S3).

CDR L1 in h-5E5 is among the longest that had been structurally characterized, and it does assume a canonical conformation in h-5E5 (Al-Lazikani *et al.*, 1997 ▸). The L1 CDR is stabilized internally by three main-chain hydrogen bonds in an antiparallel β-sheet, and four side chain–main chain and side chain–side chain intramolecular hydrogen bonds contribute to L1 having among the lowest temperature factors in the protein, with an average of 14 Å^2^ compared with an overall average of 16 Å^2^ for the light chain and 17 Å for the heavy chain.

The structures of the CDR L1 loops on the murine Fvs are all similar to h-5E5, with a few C^α^ positions at the tip of the loop showing a maximum difference of less than 1.5 Å after superposition of the Vl domains.

It was interesting to observe that the Fabs of h-5E5 pack head-to-head about a crystallographic twofold axis. Although the surfaces between the two Fabs are largely complementary, no direct interactions between them are observed outside those involving CDR L1 (Fig. 1 ▸, Table 2 ▸). The two Fabs nevertheless form a solvent-excluded interface containing 44 buried water molecules, two of which bridge between the L1 CDR of one Fab and H2 CDR of the symmetry-related Fab (Fig. 1 ▸*a* and 1 ▸*b*). The total solvent-excluded surface area was calculated to be 858 Å^2^ using the Connolly algorithm (Connolly, 1983 ▸) with the buried water molecules removed from the structure.

### 4.2. H-5E5 Fab and mAb 5E5 do not exhibit homophilic binding

The packing of the L1 CDR of a neighboring molecule into the combining site of h-5E5 so as to occlude the paratope was a potential explanation for the lack of the observation of a complex structure with the antigen; however, SPR studies showed no significant homophilic interaction for both the h-5E5 Fab (Fig. 3 ▸*a*) and mAb 5E5 (Fig. 3 ▸*b*) and occlusion of the antigen may stem in part from crystal-packing forces.

Some prominent examples of Fabs shown to crystallize via head-to-head packing are given in Supplementary Table S1. Most have their Ab combining sites positioned directly head-to-head, with engagement of CDRs from both chains, although examples exist where the interaction is offset [such as mAb R24 (PDB entry 1r24) in Supplementary Table S1]. One well known structure is the Fab from mouse mAb GH1002 (PDB entry 1ghf), which is an anti-anti-idiotope that binds to a copy of itself (Ban *et al.*, 1996 ▸). This structure generated significant interest at the time as a structural demonstration of Jerne’s theory of idiotypic regulation of the immune system (Ban *et al.*, 1996 ▸; before the theory itself was ultimately abandoned). The Ab was selected by its ability to bind (and immunologically mimic) the anti-idiotype, and so the observation of such a strong self-idiotope was particularly interesting, and the two combining sites formed 12 hydrogen bonds with a solvent-excluded area of 2020 Å^2^.

In about half of the examples the interaction in the crystal lattice is mediated through crystallographic symmetry, as in h-5E5 (Supplementary Table S1), where both Abs make identical contributions. In others, the interactions are through non-symmetry-related molecules. The degree of complementarity varies significantly, as measured by the total number of hydrogen bonds formed and the concomitant solvent-excluded area.

Relatively few of these reports have characterized the strength of any such homophilic association, with one being Ab R24 (Kaminski *et al.*, 1999 ▸). R24 was under investigation for its specificity for ganglioside GD3, which is overexpressed on some tumor cells. The self-idiotope was characterized in the crystal structure as forming an intermolecular antiparallel β-pleated sheet consisting of eight strong hydrogen bonds, with an association strength measured by SPR as 18 n*M* (*K*_D_; Kaminski *et al.*, 1999 ▸).

In terms of the number of strong hydrogen bonds and the calculated buried surface area, the interaction observed here between Fabs of h-5E5 is among the smaller self-associations reported, with six hydrogen bonds and 858 Å^2^, but given the apparent association in the lattice it is nevertheless interesting that no homophilic binding could be detected.

### 4.3. The six crystallographically independent molecules of murine 5E5 Fv provide an excellent base for comparison

The 3.0 Å resolution structure of the murine 5E5 scFv has been reported in complex with the singly glycosylated peptide APGST*AP (Macías-León *et al.*, 2020 ▸). Despite its relatively low reported resolution, this is a fascinating structure containing six independent molecules in the asymmetric unit. Five of the six molecules are reportedly observed in complex with the ligand or some portion of the ligand, with only molecule VI observed not to be in complex. Perhaps not surprisingly for the 3.0 Å resolution structure, the temperature factors for the main-chain atoms of the 12 polypeptide chains are quite high (averaging from 77 to 127 Å^2^), which indicates a large amount of motion and/or disorder in the crystal. However, and as reported, there exists appropriate electron density for most main-chain atoms (although a few of the molecules are missing sections of polypeptide).

### 4.4. Murine 5E5 Fvs display stable domain association

While comparisons of side-chain positions among structures reported to 3.0 Å resolution must be performed with caution, the relative positions of the heavy- and light-chain domains themselves (*i.e.* the ‘domain association’) can be performed with confidence as it relies on the superposition of approximately 100 amino-acid residues. The interface between Vh and Vl domains is universally composed of hydrophobic surfaces and has never been observed to contain any main chain–main chain hydrogen bonds, which contributes to the Vh–Vl interface being somewhat labile (Chailyan *et al.*, 2011 ▸). For example, it is common to see a displacement of these domains between the liganded and unliganded Ab structures (Haji-Ghassemi *et al.*, 2014 ▸). The conformational flexibility of Vh–Vl domain association has been postulated to increase the capacity of the Ab repertoire to recognize antigens (Fernández-Quintero *et al.*, 2020 ▸). However, in considering any given Ab, any domain movement upon antigen binding would be expected to impact the energetics of binding, and a number of studies have detailed the importance of residues lining the Vh–Vl interface for stable domain association and, ultimately, antigen binding (Chothia *et al.*, 1998 ▸; Vargas-Madrazo & Paz–García, 2003 ▸; Abhinandan & Martin, 2010 ▸).

To avoid the effect of the superposition being overly affected by gross changes in the conformation of small sections of polypeptide, shifts in domain association in Table 3 ▸ and Supplementary Table S2 were calculated using superposition of C^α^ atoms via the *PyMOL* (Schrödinger, 2023 ▸) SUPER function that employs an iterative process where, after each overlap cycle, corresponding C^α^ pairs that are separated by a statistically significant distance are excluded from subsequent cycles. In this way, upon convergence of this process, a structurally conserved core is identified for each pair of overlapped proteins which can be used to calculate accurately not only the similarity of the domain pairs, but also their relative movement from crystal structure to crystal structure to give a direct measure of any change in domain association.

Further, and perhaps more significantly, the identification of the C^α^ atoms in these structurally conserved cores serves to identify the conservation of gross features in the domains and can shed light on the overall effect of the humanization process: that is, the superposition algorithm will reveal whether the humanization process has disrupted one section of the domain core to a greater extent than the rest, so that if the common core structures of the Vh and/or Vl domains have not been disrupted between the parent (usually murine) and humanized structures, a greater fraction of C^α^ atoms will be identified as part of a conserved core structure.

Table 3 ▸ shows the root-mean-square deviations (r.m.s.d.s) of the core C^α^ positions of all pairwise combinations (murine and murine as well as murine and humanized) of Vh and Vh domains and of Vl and Vl domains after their least-squares superposition. In addition, after identifying the structurally conserved cores of each Vh domain, their respective r.m.s.d. after superposition of the corresponding Vl domain is also given. It is this number that represents the domain shift. The number of C^α^ atoms used in each calculation varies firstly because some of the murine models are incomplete and secondly because there are minor differences in conformation among the murine Fvs that result in some corresponding C^α^ atoms being too far apart to be considered part of their common core. The core atoms of the murine Vh domain used for comparison with the humanized Vh domain were from molecule II, as the authors had suggested that molecule II was the best-determined molecule of the murine structure (Macías-León *et al.*, 2020 ▸).

Table 3 ▸ gives a number of interesting results. Firstly, the consistently low deviations among the murine Vl and murine Vh core-domain structures, which are 0.096 ± 0.015 and 0.122 ± 0.027 Å. Secondly, the r.m.s.d. among the Vh-domain positions arising from superposition of the Vl domains, which again is a direct measure of the relative motions of the Vh and Vl domains about their common interface, is also consistent although significantly higher at 0.77 ± 0.34 Å (revealing the relative lability of the domain association). Further, Table 3 ▸ shows that domain association in this Ab does not change markedly upon complexation, as the values for molecule VI (which was observed not to be liganded) are similar to those of the other five Fvs that are observed to be in complex with ligand.

### 4.5. Humanized 5E5 shows a distinct domain shift from murine 5E5 Fv

The rightmost data column in Table 3 ▸ contains the r.m.s.d.s of the corresponding overlaps of the core C^α^ atoms of the humanized Fv with all six Fvs in the asymmetric unit of the murine structure. The modest variation in both core structures (0.096 ± 0.015 Å for 83–100 C^α^ atoms for the Vl domain and 0.122 ± 0.027 Å for 89–107 C^α^ atoms for the Vh domain) compared with the spread in domain association (∼0.77 ± 0.34 Å) of the six independent molecules in the murine 5E5 Fv structures allows some insight into the effects of the humanization process.

Firstly, the conservation of the core structures of the humanized Vl and Vh domains is indicated by the r.m.s.d. from the corresponding murine structures, which is 0.31 ± 0.02 and 0.31 ± 0.012 Å. Poljak established in a landmark paper some 35 years ago that the presence of the first constant domains tethered to the Fv domains through the flexible elbow regions (Stanfield *et al.*, 2006 ▸) do not influence binding affinity significantly (Bhat *et al.*, 1990 ▸), and so the observed deviations in the C^α^ cores of h-5E5 of 2.5–3 times that of the corresponding murine domains among themselves must be attributed to the humanization process.

Secondly, the shift in domain association is given by the r.m.s.d.s of the core Vh C^α^ atoms after superposition of the core Vl C^α^ atoms, which range from 0.93 to 1.65 Å with an average of 1.33 ± 0.28 Å: about twice the average r.m.s.d. observed in domain shift among the six murine 5E5 Fv structures. This indicates that the h-5E5 interface has been somewhat affected, which might impinge on antigen binding. For example, the significant 1.59 Å shift in domain association around the combining site between the Vh domain of molecule II of the murine structure with the Vh domain of the humanized Fab is easily seen in Fig. 1 ▸(*d*).

For comparison, the corresponding calculations were carried out on three well known structurally characterized pairs of murine parent and corresponding humanized Abs (Supplementary Table S2). The first example concerns the humanized Ab (PDB entry 3aaz) of murine mAb WO-2 (PDB entry 3bae) specific for the Aβ peptide implicated in Alzheimer’s disease (Robert *et al.*, 2010 ▸). The humanized mAb hWO-2 showed an approximate sixfold drop in affinity over the murine parent while exhibiting a domain shift of 1.45 Å. Interestingly, in calculating the overlapped core structures, *PyMOL* (Schrödinger, 2023 ▸) excluded 21 C^α^ atoms from the 116-atom heavy chain and 14 C^α^ atoms from the 113-atom light chain, which indicates that parts of the core structures of the two domains have been shifted as a result of the humanization.

The second example is the humanized Ab (PDB entry 1it9) of the murine anti-Fas Ab HFE7A (PDB entry 1iqw; Ito *et al.*, 2002 ▸), which displayed binding affinity on the same order as the parent murine Ab and which shows a domain shift of the core C^α^ atoms of 0.82 Å. Again interestingly, the core structure excludes only 11 C^α^ atoms from the 121-C^α^-atom heavy chain but 25 C^α^ atoms from the 112-C^α^-atom light chain, showing that the humanization of the heavy chain has succeeded in largely replicating the backbone structure of the parent murine heavy chain.

The third example, and perhaps the most significant one to this study, is the recently reported humanization of the murine Ab specific for Pan-HLA-DR mAb44H10 (PDB entry 8euq; Kassardjian *et al.*, 2024 ▸), which describes a tour de force of structure-guided humanization that includes the crystal structures of three initial attempts at humanization (PDB entries 9b74, 9b75 and 9b76) that displayed relatively poorer activity towards the target antigen as well as the structure of the final humanized Ab (PDB entry 9b7b) that shows affinity approaching that of the parent murine Ab (PDB entry 8euq; Kassardjian *et al.*, 2023 ▸). In refining the humanization process through a comparison of the structures of the preliminary attempts with the structure of the parent the authors demonstrate that the final humanized clone also displays nanomolar binding.

In correspondence with the high binding of the humanized antibody, there is a remarkable correspondence of its core residues with those of the parent (Supplementary Table S2). The domain shifts modestly improved from about 0.9 Å to about 0.7 Å, but while the number of C^α^ atoms included in the light-chain core was largely unchanged, the number of C^α^ atoms included in the heavy-chain core included all but four C^α^ atoms, going from 96 to 116 of 120 C^α^ atoms.

### 4.6. Implications for the humanization of 5E5

The domain shift of h-5E5 from the parent murine Fvs is about 1.33 Å, which is about double the average domain shift observed among the six molecules of the murine 5E5. Further, in comparing with their gross structures, the exclusion of 20–25 C^α^ atoms from the core in both the heavy and light chains during the superposition process indicates that the humanization process has introduced significant change into the core structure. Of the 24 residues corresponding to the C^α^ atoms excluded from the core in the comparison between molecule II and h-5E5, fully 20 of them in both the heavy and light chains are either humanizing mutations or within 5 Å of a humanizing mutation. As well, 20 of the 37 total humanizing mutations lie within 10 Å of a combining site residue and would have the potential to affect binding (Wedemayer *et al.*, 1997 ▸).

## 5. Related literature

The following references are cited in the supporting information for this article: Haruyama *et al.* (2002 ▸), Larson *et al.* (2005 ▸), Miles *et al.* (2008 ▸) and Trakhanov *et al.* (1999 ▸).

## Supplementary Material

PDB reference: humanized 5E5 Fab, 9eci

Supplementary Tables and Figures. DOI: 10.1107/S2059798325002554/ag5055sup1.pdf

## Figures and Tables

**Table 1:** Data-collection and refinement statistics for the humanized 5E5 Fab

Data collection	
Space group	*C*121
Resolution (Å)	50.00–1.57 (1.60–1.57)
Temperature (K)	100
*a*, *b*, *c* (Å)	102.24, 75.34, 69.65
α, β, γ (°)	90, 106.28, 90
*Z*	1
*R*_merge_	0.103 (0.331)
*R*_p.i.m._	0.037 (0.201)
CC_1/2_	0.970 (0.895)
〈*I*/σ(*I*)〉	17.4 (2.0)
Completeness (%)	99.9 (99.5)
Multiplicity	6.6 (3.6)
Total reflections	468879
Unique reflections	70587
Refinement	
Resolution (Å)	33.43–1.57
No. of reflections	69451
*R*_work_ (%)	0.1547
*R*_free_ (%)	0.1819
No. of atoms	
Protein	3494
Ligand	8 [MPD†]
Water	768
*B* factors (Å^2^)	
Protein	16.1
Water	29.8
Average	18.6
Ramachandran statistics	
Favored (%)	98.1
Allowed (%)	1.9
R.m.s.d., bond lengths (Å)	0.006
R.m.s.d., angles (°)	0.86

† 2-Methyl-2,4-pentanediol [CH_3_CH(OH)CH_2_C(CH_3_)_2_OH].

**Table 2:** Unique hydrogen bonds formed between the combining sites of two Fabs in the crystal lattice The long L1 CDR from one Fab extends into the combining site of the crystallographically related neighbor and vice versa to form six strong hydrogen bonds directly linking the pair of Fabs.

Fab (*x*, *y*, *z*)		Fab (−*x*, *y*, −*z*)		Distance (Å)
Ser32 OH	L1	Asp57 OD2	H2	2.3
Gly33 O	L1	Thr100 OG1	H3	2.9
Gln35 NE2	L1	Ser52 OG	H2	2.9

**Table 3:** Comparison of the r.m.s.d.s for the superposed Vh domain cores, the superposed Vl domain cores and the Vh domain core after superposition of the corresponding Vl domain core for each of the six observed Fv structures of murine 5E5 (Macías-León *et al.*, 2020 ▸; I–VI from PDB entry 6tnp) and h-5E5 (H), as defined by the *PyMOL* suite (Schrödinger, 2023 ▸) least-squares overlap routines Values in parentheses are the number of atoms used in each r.m.s.d. calculation.

	I	II	III	IV	V	VI	〈av〉	H	
I	—	0.114 (98)	0.104 (98)	0.122 (99)	0.104 (99)	0.080 (87)	0.104	0.286 (92)	Vh
		0.097 (94)	0.106 (101)	0.123 (93)	0.159 (97)	0.135 (102)	0.124	0.313 (91:21)	Vl
		0.521 (98)	0.711 (98)	0.574 (96)	1.139 (98)	0.467 (87)	0.682	1.646 (91:24)	Vh (Vl)
II	0.114 (98)	—	0.063 (89)	0.087 (93)	0.092 (96)	0.093 (84)	0.088	0.298 (91)	Vh
	0.097 (94)		0.094 (100)	0.081 (89)	0.165 (103)	0.114 (107)	0.110	0.312 (88:24)	Vl
	0.521 (98)		0.794 (98)	0.254 (95)	1.363 (98)	0.495 (87)	0.685	1.585 (91:24)	Vh (Vl)
III	0.104 (98)	0.063 (89)	—	0.104 (100)	0.079 (97)	0.095 (85)	0.088	0.311 (94)	Vh
	0.106 (101)	0.094 (100)		0.117 (97)	0.146 (101)	0.096 (106)	0.112	0.304 (90:22)	Vl
	0.711 (98)	0.794 (98)		0.895 (95)	0.438 (87)	1.125 (98)	0.793	0.983 (91:24)	Vh (Vl)
IV	0.122 (99)	0.087 (93)	0.104 (100)	—	0.107 (93)	0.073 (83)	0.098	0.341 (91)	Vh
	0.123 (93)	0.081 (89)	0.117 (97)		0.172 (95)	0.096 (94)	0.118	0.313 (87:25)	Vl
	0.574 (96)	0.254 (95)	0.895 (95)		1.374 (95)	0.554 (87)	0.730	1.516 (88:21)	Vh (Vl)
V	0.103 (99)	0.092 (96)	0.079 (97)	0.105 (93)	—	0.101 (83)	0.096	0.300 (95)	Vh
	0.159 (97)	0.165 (103)	0.146 (101)	0.172 (95)		0.127 (97)	0.154	0.315 (92:20)	Vl
	1.139 (98)	1.363 (98)	0.438 (87)	1.374 (95)		0.909 (87)	1.045	0.934 (91:24)	Vh (Vl)
VI	0.078 (87)	0.088 (84)	0.091 (85)	0.071 (83)	0.099 (83)	—	0.085	0.321 (80)	Vh
	0.135 (102)	0.114 (107)	0.096 (106)	0.096 (94)	0.127 (97)		0.114	0.280 (80:21)	Vl
	0.467 (87)	0.495 (87)	1.125 (98)	0.554 (87)	0.909 (87)		0.710	1.312 (80:21)	Vh (Vl)
〈av〉	0.104	0.088	0.088	0.098	0.096	0.085	0.093	0.305	Vh
	0.124	0.110	0.112	0.118	0.154	0.114	0.122	0.306	Vl
	0.682	0.685	0.793	0.730	1.045	0.710	0.774	1.329	Vh (Vl)
