# Supplementary material for: Structure of the Fab fragment of a humanized 5E5 antibody to a cancer-specific Tn-MUC1 epitope
Source: Acta Crystallogr D Struct Biol. 2025 Apr 13;81(Pt 5):223–33. doi: 10.1107/S2059798325002554 (PMC12054364; doi:10.1107/S2059798325002554)
Supplement: Supplementary file 1 [file d-81-00223-sup1.pdf]

STRUCTURAL BIOLOGY

Volume 81 (2025)

Supporting information for article:

Structure of the Fab fragment of a humanized 5E5 antibody to a cancer-specific Tn-MUC1 epitope

Wei Li, Ulla Mandel, Henk van Faassen, Matthew J. Parker, Max S. G. Legg, Greg Hussack, Henrik Clausen and Stephen V. Evans

Table S1 Other published structures of Fabs that display head-to-head packing

PDB ID	# H-bonds	Solvent excluded area (Å^2)	2-fold	*Z*	Notes
9ECI[†]	6	858	Y	1	1 molecule in the AU related to another by a 2-fold axis
1ZA6 (Larson *et al.*, 2005)	6	1125	N	4	4 molecules in the AU forming 2 head-to-head arrangements with nearly identical interactions
6PE7 (Argiriadi *et al.*, 2019)	13	1674	Y	1	2 combining sites arranged about a 2-fold axis. Tyr-69 forms a symmetry-related contact with its corresponding residue.
12E8 (Trakhanov *et al.*, 1999)	12	1301	N	2	2 molecules in the AU approximately related by a non-crystallographic 2-fold axis
1R24 (Kaminski *et al.*, 1999)	8	561	N	2	2 molecules in the AU sharing a β-sheet interaction along one side of the combining site away from the paratope.
1GHF (Ban *et al.*, 1996)	12	2020	Y	1	1 molecule in the AU related to its head-to-head partner by a 2-fold axis

[†]Current study.

Table S2 . Comparison of the corresponding RMS deviations in the VH domain core and VL domain core after least-squares superposition of the 'core' VL domains*

Superposed Humanized Domain		Reference Murine Domain			
		VH deviations (core:excluded)		VL deviations (core:excluded)	
		3BAE(Miles *et al.*, 2008)			
3AAZ (Robert *et al.*, 2010)	VH (core)	0.443	(95:21)		
	VL (core)	1.450	(95:21)	0.424	(99:14)
		1IQW(Ito *et al.*, 2002)			
1IT9 (Haruyama *et al.*, 2002)	VH (core)	0.331	(110:11)		
	VL (core)	0.819	(110:11)	0.319	(87:25)
		8EUQ(Kassardjian *et al.*, 2023)			
9B74 (Kassardjian *et al.*, 2024)	VH (core)	0.387	(96:24)		
	VL (core)	0.855	(96:24)	0.466	(98:10)
9B75 (Kassardjian *et al.*, 2024)	VH (core)	0.520	(106:13)		
	VL (core)	0.914	(106:13)	0.428	(96:12)
9B76 (Kassardjian *et al.*, 2024)	VH (core)	0.343	(104:16)		
	VL (core)	0.683	(104:16)	0.408	(97:11)
9B7B (Kassardjian *et al.*, 2024)	VH (core)	0.370	(116:4)		
	VL (core)	0.686	(116:4)	0.442	(96:12)

*As defined by the PyMOL suite least-squares overlap routines. Numbers in parentheses are the number of atoms used in each RMS calculation and the number of atoms excluded from the calculation.

```
SEQ_1      QVQLQQSDAELVKPGSSVKISCKASGYTFTDHAIHWVKQKPEQGLEW-IGHFSPGNTDIK    59
SEQ_2      QVQLVQSGAEVKKTGSSVKVSCKASGYTFTDHAIHWVRQAPGQALEVVMGHFSPGNTDIK    60
SEQ_3      QVQLVQSGAEVKKTGSSVKVSCKASGYTFTDHAIHWVRQAPGQALEW-IGHFSPGNTDIK    59
SEQ_4      QVQLVQSGAEVKKTGSSVKVSCKASGYTFTDHAIHWVRQAPGQALEVVMGHFSPGNTDIK    60
SEQ_5      QVQLVQSGAEVKKTGSSVKVSCKASGYTFTDHAIHWVRQAPGQALEW-IGHFSPGNTDIK    59
SEQ_6      QVQLVQSGAEVKKTGSSVKVSCKASGYTFTDHAIHWVRQAPGQALEW-IGHFSPGNTDIK    59
SEQ_7      QVQLVQSGAEVKKTGSSVKVSCKASGYTFTDHAIHWVRQAPGQALEW-IGHFSPGNTDIK    59
SEQ_8      QVQLVQSGAEVKKTGSSVKVSCKASGYTFTDHAIHWVRQAPGQALEW-IGHFSPGNTDIK    59
SEQ_9      QVQLVQSGAEVKKTGSSVKVSCKASGYTFTDHAIHWVRQAPGQALEVVMGHFSPGNTDIK    60
SEQ_10     EVQLVQSGAEVKKPGESLKISCKISGYIFTDHAIHWVRQMPGKGLEVVMGHFSPGNTDIK    60
SEQ_11     EVQLVQSGAEVKKPGESLKISCKISGYTFTDHAIHWVRQMPGKGLEW-IGHFSPGNTDIK    59
SEQ_12     EVQLVQSGAEVKKPGESLKISCKISGYIFTDHAIHWVRQMPGKGLEW-IGHFSPGNTDIK    59
SEQ_13     EVQLVQSGAEVKKPGESLKISCKISGYTFTDHAIHWVRQMPGKGLEW-MGHFSPGNTDIK    59
SEQ_14     EVQLVQSGAEVKKPGESLKISCKISGYTFTDHAIHWVRQMPGKGLEW-IGHFSPGNTDIK    59
SEQ_15     EVQLVQSGAEVKKPGESLKISCKISGYTFTDHAIHWVRQMPGKGLEW-IGHFSPGNTDIK    59
SEQ_16     EVQLVQSGAEVKKPGESLKISCKISGYTFTDHAIHWVRQMPGKGLEW-IGHFSPGNTDIK    59
SEQ_17     EVQLVQSGAEVKKPGESLKISCKISGYTFTDHAIHWVRQMPGKGLEW-IGHFSPGNTDIK    59
SEQ_18     EVQLVQSGAEVKKPGESLKISCKISGYIFTDHAIHWVRQMPGKGLEVVMGHFSPGNTDIK    60
SEQ_19     EVQLVQSGAEVKKPGESLKISCKISGYIFTDHAIHWVRQMPGKGLEVVMGHFSPGNTDIK    60
SEQ_20     EVQLVESGAEVKKPGASVEVSCQASGYTFTDHAIHWVRQAPGQGLEW-MGHFSPGNTDIK    59
SEQ_21     EVQLVESGAEVKKPGASVEVSCQASGYTFTDHAIHWVRQAPGQGLEW-IGHFSPGNTDIK    59
SEQ_22     EVQLVESGAEVKKPGASVEVSCQASGYTFTDHAIHWVRQAPGQGLEW-MGHFSPGNTDIK    59
SEQ_23     EVQLVESGAEVKKPGASVEVSCQASGYTFTDHAIHWVRQAPGQGLEW-IGHFSPGNTDIK    59
SEQ_24     EVQLVESGAEVKKPGASVEVSCQASGYTFTDHAIHWVRQAPGQGLEW-IGHFSPGNTDIK    59
SEQ_26     EVQLVESGAEVKKPGASVEVSCQASGYTFTDHAIHWVRQAPGQGLEW-IGHFSPGNTDIK    59
SEQ_27     EVQLVESGAEVKKPGASVEVSCQASGYTFTDHAIHWVRQAPGQGLEW-IGHFSPGNTDIK    59
SEQ_28     EVQLVESGAEVKKPGASVEVSCQASGYTFTDHAIHWVRQAPGQGLEW-MGHFSPGNTDIK    59
SEQ_29     EVQLVESGAEVKKPGASVEVSCQASGYTFTDHAIHWVRQAPGQGLEW-MGHFSPGNTDIK    59
           :*** :*.**: * * *:::**: *** *********:* * :.**  :***********
```

```
SEQ_1     YNDKFKGKATLTVDRSSSTAYMQLNSLTSEDSAVYFCKTSTFFFDYWGQGTTLTVSS   116
SEQ_2     YNDKFKGRVTITRDRSMSTAYMELSSLRSEDTAMYYCATSTFFFDYWGQGTMVTVSS   117
SEQ_3     YNDKFKGRATLTVDRSMSTAYMELSSLRSEDTAMYYCKTSTFFFDYWGQGTMVTVSS   116
SEQ_4     YNDKFKGRATLTVDRSMSTAYMELSSLRSEDTAMYYCKTSTFFFDYWGQGTMVTVSS   117
SEQ_5     YNDKFKGRVTLTVDRSMSTAYMELSSLRSEDTAMYYCKTSTFFFDYWGQGTMVTVSS   116
SEQ_6     YNDKFKGRATITVDRSMSTAYMELSSLRSEDTAMYYCKTSTFFFDYWGQGTMVTVSS   116
SEQ_7     YNDKFKGRATLTRDRSMSTAYMELSSLRSEDTAMYYCKTSTFFFDYWGQGTMVTVSS   116
SEQ_8     YNDKFKGRATLTVDRSMSTAYMELSSLRSEDTAMYYCATSTFFFDYWGQGTMVTVSS   116
SEQ_9     YNDKFKGRVTLTVDRSMSTAYMELSSLRSEDTAMYYCKTSTFFFDYWGQGTMVTVSS   117
SEQ_10    YNDKFKGQVTFSVDRSINTAYLQWSSLKASDTAIYFCARSTFFFDYWGQGTRVTVSS   117
SEQ_11    YNDKFKGQATLSVDRSINTAYLQWSSLKASDTAIYFCKTSTFFFDYWGQGTRVTVSS   116
SEQ_12    YNDKFKGQATLSVDRSINTAYLQWSSLKASDTAIYFCKTSTFFFDYWGQGTRVTVSS   116
SEQ_13    YNDKFKGQATLSVDRSINTAYLQWSSLKASDTAIYFCKTSTFFFDYWGQGTRVTVSS   116
SEQ_14    YNDKFKGQVTLSVDRSINTAYLQWSSLKASDTAIYFCKTSTFFFDYWGQGTRVTVSS   116
SEQ_15    YNDKFKGQATFSVDRSINTAYLQWSSLKASDTAIYFCKTSTFFFDYWGQGTRVTVSS   116
SEQ_16    YNDKFKGQATLSVDRSINTAYLQWSSLKASDTAIYFCATSTFFFDYWGQGTRVTVSS   116
SEQ_17    YNDKFKGQATLSVDRSINTAYLQWSSLKASDTAIYFCKRSTFFFDYWGQGTRVTVSS   116
SEQ_18    YNDKFKGQATLSVDRSINTAYLQWSSLKASDTAIYFCKTSTFFFDYWGQGTRVTVSS   117
SEQ_19    YNDKFKGQVTLSVDRSINTAYLQWSSLKASDTAIYFCKTSTFFFDYWGQGTRVTVSS   117
SEQ_20    YNDKFKGRVTMTRDTSINTAYMELRRLRSDDTAVYYCATSTFFFDYWGQGTMVTVSS   116
SEQ_21    YNDKFKGRATLTVDRSINTAYMELRRLRSDDTAVYYCKTSTFFFDYWGQGTMVTVSS   116
SEQ_22    YNDKFKGRATLTVDRSINTAYMELRRLRSDDTAVYYCKTSTFFFDYWGQGTMVTVSS   116
SEQ_23    YNDKFKGRVTLTVDRSINTAYMELRRLRSDDTAVYYCKTSTFFFDYWGQGTMVTVSS   116
SEQ_24    YNDKFKGRATMTVDRSINTAYMELRRLRSDDTAVYYCKTSTFFFDYWGQGTMVTVSS   116
SEQ_26    YNDKFKGRATLTVDTSINTAYMELRRLRSDDTAVYYCKTSTFFFDYWGQGTMVTVSS   116
SEQ_27    YNDKFKGRATLTVDRSINTAYMELRRLRSDDTAVYYCATSTFFFDYWGQGTMVTVSS   116
SEQ_28    YNDKFKGRVTMTVDRSINTAYMELRRLRSDDTAVYYCKTSTFFFDYWGQGTMVTVSS   116
SEQ_29    YNDKFKGRATMTVDRSINTAYMELRRLRSDDTAVYYCKTSTFFFDYWGQGTMVTVSS   116
          *******:.*:: * * .***::   * :.*:*:*:*  ************ :****
```

A.

```
SEQ_30    DIVMTQSPSSLTVTAGEKVTMICKSSQSLLNSGDQKNYLTWYQQKPGQPPKLLIFWASTR   60
SEQ_31    DIVMTQSPDSLAVSLGERATINCKSSQSLLNSGDQKNYLTWYQQKPGQPPKLLIYWASTR   60
SEQ_32    DIVMTQSPDSLAVSLGERATIICKSSQSLLNSGDQKNYLTWYQQKPGQPPKLLIFWASTR   60
SEQ_33    DIVMTQSPLSLPVTPGEPASISCKSSQSLLNSGDQKNYLTWYLQKPGQSPQLLIYWASTR   60
SEQ_34    DIVMTQSPLSLPVTPGEPASIICKSSQSLLNSGDQKNYLTWYLQKPGQSPQLLIFWASTR   60
          ******** ** *: ** .:: ******************** ***** *:***:*****

SEQ_30    ESGVPDRFTGSGSGTDFTLTISSVQAEDLAVYYCQNDYSYPLTFGAGTKLELK 113
SEQ_31    ESGVPDRFSGSGSGTDFTLTISSLQAEDVAVYYCQNDYSYPLTFGQGTKVEIK 113
SEQ_32    ESGVPDRFTGSGSGTDFTLTISSLQAEDVAVYYCQNDYSYPLTFGQGTKVEIK 113
SEQ_33    ESGVPDRFSGSGSGTDFTLKISRVEAEDVGVYYCQNDYSYPLTFGGGTKVEIK 113
SEQ_34    ESGVPDRFTGSGSGTDFTLKISRVEAEDVGVYYCQNDYSYPLTFGGGTKVEIK 113
          ********:**********.** ::***:.*************** ***:*:*
```

B.

Figure S1 Generated humanized clones of murine 5E5. **A.** The murine Fv heavy chain sequence (SEQ_1) compared to 27 of 28 generated heavy chain clones. SEQ_5 (bold) clone most closely resembles the identified sequence of h-5E5, with the exception being I48M. SEQ_25 is not included in alignment as it differs significantly. **B.** The murine light chain sequence (SEQ_30) compared to all 4 generated light chain clones (SEQ_31-SEQ_34). H-5E5 is SEQ_31.

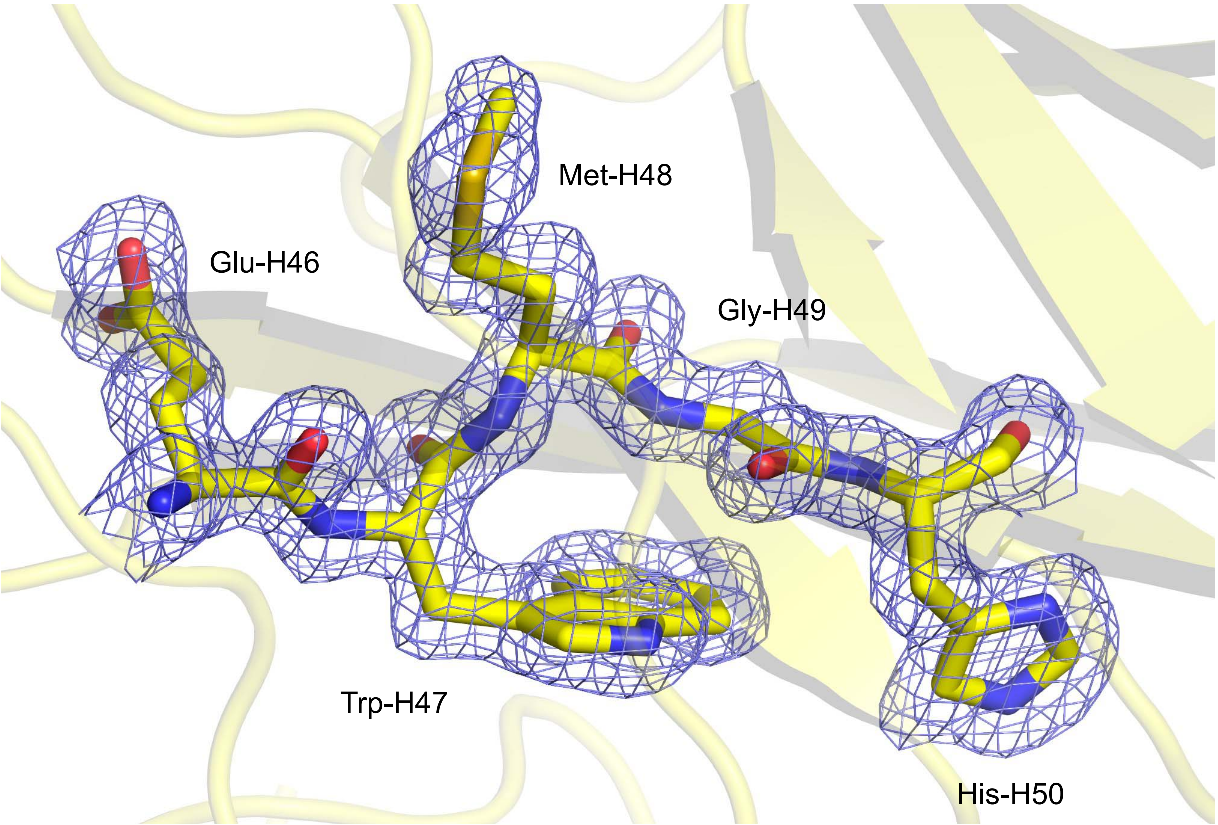

Figure S2 The electron density surrounding heavy chain residue 48 unambiguously corresponds to a methionine residue instead of the expected isoleucine of SEQ_5. The 2Fo-Fc electron density map was contoured at 1.0 σ.

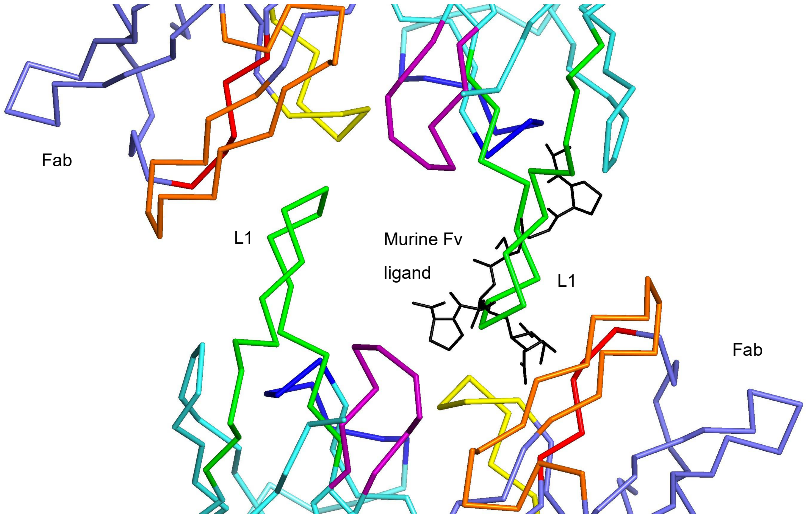

Figure S3 Wireframe model of the α-carbon trace of the h-5E5 combining site, showing the insertion of the L1 loop into the combining site of a neighboring Fab. While the residues involved in forming hydrogen bonds are not the same ones used to bind antigen in the murine Fv structure, the loops do occlude the putative paratope, as demonstrated by the superposition of h-5E5 with the murine Fv (not shown). Murine ligand (APGST*AP), black; H-5E5 L1, green.

References

Argiriadi, M. A., Benatuil, L., Dubrovska, I., Egan, D. A., Gao, L., Greischar, A., Hardman, J., Harlan, J., Iyer, R. B. & Judge, R. A. (2019). *BMC molecular and cell biology* **20**, 1-13.

Ban, N., Day, J., Wang, X., Ferrone, S. & McPherson, A. (1996). *Journal of molecular biology* **255**, 617-627.

Haruyama, H., Ito, S., Miyadai, K., Takahashi, T., Kawaida, R., Takayama, T., Hanzawa, H., Hata, T., Yamaguchi, J. & Yoshida-Kato, H. (2002). *Biological and Pharmaceutical Bulletin* **25**, 1537-1545.

Ito, S., Takayama, T., Hanzawa, H., Ichikawa, K., Ohsumi, J., Serizawa, N., Hata, T. & Haruyama, H. (2002). *The journal of biochemistry* **131**, 137-143.

Kaminski, M. J., MacKenzie, C. R., Mooibroek, M. J., Dahms, T. E., Hirama, T., Houghton, A. N., Chapman, P. B. & Evans, S. V. (1999). *Journal of Biological Chemistry* **274**, 5597-5604.

Kassardjian, A., Ivanochko, D., Barber, B., Jetha, A. & Julien, J.-P. (2024). *Antibodies* **13**, 57.

Kassardjian, A., Sun, E., Sookhoo, J., Muthuraman, K., Boligan, K. F., Kucharska, I., Rujas, E., Jetha, A., Branch, D. R. & Babiuk, S. (2023). *Cell Reports* **42**,

Larson, S. B., Day, J. S., Glaser, S., Braslawsky, G. & McPherson, A. (2005). *Journal of molecular biology* **348**, 1177-1190.

Miles, L. A., Wun, K. S., Crespi, G. A., Fodero-Tavoletti, M. T., Galatis, D., Bagley, C. J., Beyreuther, K., Masters, C. L., Cappai, R. & McKinstry, W. J. (2008). *Journal of molecular biology* **377**, 181-192.

Robert, R., Streltsov, V. A., Newman, J., Pearce, L. A., Wark, K. L. & Dolezal, O. (2010). *Protein Science* **19**, 299-308.

Trakhanov, S., Parkin, S., Raffaï, R., Milne, R., Newhouse, Y. M., Weisgraber, K. H. & Rupp, B. (1999). *Acta Crystallographica Section D: Biological Crystallography* **55**, 122-128.
